# Osteopontin Regulates Treg Cell Stability and Function with Implications for Anti-Tumor Immunity and Autoimmunity

**DOI:** 10.3390/cancers16172952

**Published:** 2024-08-24

**Authors:** Aigli G. Vakrakou, Evangelia Kourepini, Ioannis Skordos, Natalia Nieto, Vily Panoutsakopoulou, Nikolaos Paschalidis

**Affiliations:** 1 Laboratory of Neuroimmunology, First Department of Neurology, Aeginition Hospital, National and Kapodistrian, University of Athens, 21 Papadiamantopoulou, Ilisia, 11528 Athens, Greece; avakrakou@med.uoa.gr; 2 Biomedical Research Foundation, Academy of Athens, 4 Soranou Efessiou Street, 11527 Athens, Greece; ekourep@eie.gr (E.K.); ioannis.skordos@irc.vib-ugent.be (I.S.);; 3 Department of Pathology, University of Illinois at Chicago, Chicago, IL 60612, USA; nnieto@uic.edu

**Keywords:** osteopontin, Foxp3, Treg, melanoma, colitis, flow cytometry

## Abstract

**Simple Summary:**

T regulatory cells are specialized lymphocytes that prevent excessive immune responses, yet they can suppress beneficial anti-tumor immunity when infiltrating malignant tumors. Osteopontin, a versatile protein produced by various cells, including T lymphocytes, influences both inflammation-related diseases and cancer immunity. Its role in T regulatory cells, however, remains less explored. Our study examined the impact of osteopontin deficiency on T regulatory cells. We generated mice lacking osteopontin in these cells and observed impaired immune suppression capabilities, which resulted in increased tumor-fighting activity against melanoma. These findings emphasize osteopontin’s crucial role in T regulatory cell stability and functionality within the tumor microenvironment, providing valuable insights for developing new immunotherapeutic strategies.

**Abstract:**

Foxp3-expressing regulatory T (Treg) cells represent the most highly immunosuppressive cell in the tumor microenvironment (TME) that halts effective anti-tumor immunity. Osteopontin (Opn), an extracellular matrix (ECM) glycophosphoprotein, plays key roles in many types of immune-related diseases and is associated with cancer aggressiveness when expressed by tumor cells. However, its role in Foxp3Treg heterogeneity, function, and stability in the TME is poorly defined. We generated mice with a Foxp3-specific deletion of Opn and assessed the ability of Opn-deficient Tregs to suppress inflammation. As these mice aged, they developed a scurfy-like syndrome characterized by aberrant and excessive activation of effector T cells. We evaluated and further confirmed the reduced suppressive capacity of Opn-deficient Tregs in an in vivo suppression assay of colitis. We also found that mice with Opn-deficient Foxp3^+^ Tregs have enhanced anti-tumor immunity and reduced tumor burden, associated with an unstable Treg phenotype, paralleled by reduced Foxp3 expression in tumor-infiltrating lymphocytes. Finally, we observed reduced Foxp3 and Helios expression in Opn-deficient Tregs compared to wild-type controls after in vitro activation. Our findings indicate that targeting Opn in Tregs reveals vigorous and effective ways of promoting Treg instability and dysfunction in the TME, facilitating anti-tumor immunity.

## 1. Introduction

Osteopontin (Opn), also known as early T-lymphocyte activation (ETA-1) or secreted phosphoprotein 1 (SPP1), is a pleiotropic protein with important roles in bone remodeling, chemotaxis, apoptosis, and cell activation [1]. Opn binds to integrins (αvβ3, β1, and CD49d or VLA-4) and the scavenger receptor CD44. The α4β1-OPN interaction inhibits nuclear translocation of the transcription factor forkhead box O3A (FOXO3A), negatively regulates the transcription of pro-apoptotic genes, and positively regulates the expression of anti-apoptotic genes and T-helper (Th) 1- and (Th) 17-type cytokines [2]. Opn plays a key role in the formation, maturation, differentiation, and activation of T cells: secreted Opn (sOpn) is involved in the formation of T helper type 1 (Th1) and Th17, pathogenic T cells in various autoimmune diseases, whereas intracellular Opn (iOpn) is required for the development of Th17 cells as well as the production of IFN-α [3] and IFN-β by plasmacytoid dendritic cells DCs (pDCs) [4]. iOpn is also essential for the maturation and differentiation of functional NK cells [5].

Over the past decades, studies by our group and others have highlighted the key roles of Opn in many immune-mediated diseases [6,7]. It is now evident that Opn is highly enriched in inflammatory microenvironments, especially during chronicity [8,9], and is expressed by a variety of cells of innate and adaptive immunity, including T lymphocytes [7]. Until recently, it was unknown whether Foxp3^+^ T regulatory cells (Tregs) express Opn. Our group and others have demonstrated that Opn is highly expressed in Foxp3^+^ Tregs and is involved in inducing immune tolerance against allergic diseases [6]. During the allergic airway disease phase, it is proinflammatory, whereas during the challenge phase, it exerts an anti-inflammatory role [8]. Specifically, Tregs, under the effects of Opn treatment, suppress Th2 memory and newly activated Th2 cells.

In the tumor microenvironment (TME), Opn is expressed by myeloid and tumor cells and functions as a suppressor of cytotoxic T cell activation, thus promoting host tumor immune tolerance and tumor immune evasion [10,11]. Interferon regulatory factor 8 (IRF8) in tumor cells and in CD11b + Ly6CloLy6G+ myeloid cells has been found to be a suppressor of Opn. Downregulation of IRF8 unreleased the repression of Opn, and thus, Opn could bind to the receptor CD44 on activated T cells, acting as a potent T cell suppressor [12]. Apart from acting as an immune checkpoint for T cell control, Opn favors angiogenesis and tumor progression through M2 macrophage recruitment through the secretion of prostaglandin E2 and MMP-9 from tumor-associated macrophages [13]. In addition, genomic studies by other groups have shown that the Opn gene (*Sppl*) is one of the few significantly upregulated genes in stimulated Foxp3^+^ Tregs, and tumor-infiltrating Foxp3^+^ Tregs express higher levels of *Spp1* compared to spleen and lymph node Foxp3^+^ Tregs [14,15,16]. Foxp3 Tregs infiltrate tumors, suppress beneficial anti-tumor immunity, and are associated with poor prognosis. Although Opn has been widely associated with cancer aggressiveness [17] when expressed by tumor cells, its role is uncertain when expressed by Tregs in the context of the TME.

In this study, to understand the role of Opn expression specifically in Foxp3^+^ Treg cell development, we generated mice with a Foxp3^+^ Treg-specific deletion of Opn (*Opn*^fl/fl^ *Foxp3^YFP-^*^Cre^). Our results establish a Treg-specific function for Opn in controlling stability and functionality, which significantly affects the development of inflammation and cancer cell aggressiveness.

## 2. Materials and Methods

### 2.1. Mice

The mice strains used in this study were C57BL/6J (B6), B6.SJL-*Ptprc*^a^ *Pepc*^b^/BoyJ (B6 CD45.1), B6 *Rag*2^−/−^, and B6.129(Cg)-*Foxp3*^tm4(YFP/icre)Ayr/^J (*Foxp3*^YFP-Cre^), all purchased from the Jackson Laboratory (Bar Harbor, ME, USA). The *Opn*^fl/fl^ mice were donated by Dr. Natalia Nieto (University of Illinois at Chicago, Chicago, IL, USA) [18,19]. In all experiments, we used sex- and age-matched mice between 8 and 12 weeks old, unless otherwise stated. Mice were housed and maintained at the animal facility of the Biomedical Research Foundation of the Academy of Athens (BRFAA). All mice used in the experiments were observed to be healthy and did not show any signs of abnormalities. The mice were breeding normally and exhibited no signs of an abnormal immune phenotype or other systemic issues. The *Opn*^fl/fl^*Foxp3*^YFP-Cre^ mice, as they aged (between 10 and 12 months old), began to develop signs of skin inflammation. For these mice, we ensured they received appropriate care, including more accessible food and water, and they were euthanized upon worsening of symptoms to minimize suffering. All procedures that involved the use of mice were in accordance with institutional guidelines and were approved by the Institutional Committee of Protocol Evaluation in conjunction with the Directorate of Agriculture and Veterinary Policy, Region of Attika, Greece (Protocol Approval code 1206/13-03-2018).

### 2.2. Flow Cytometry

For the analysis of T cells from *Opn*^fl/fl^*Foxp3*^YFP-Cre^ mice, thymus, LNs, and spleens were extracted, and single-cell suspensions were prepared as previously described [20]. For the staining of extracellular markers, cell suspensions were incubated with antibodies for 30 min at 4 °C. The following antibodies were used (all from BioLegend, San Diego, CA, USA): anti-CD3 FITC (145-2C11), anti-CD4 PE (GK1.5), CD8 (17A2), anti-CD44 PE-Cy7 (IM7), anti-CD62L Pacific Blue (W18021D), anti-CD45 APC-Cy7 (30-F11), anti-CTLA-4 PE (UC10-4B9), anti-ICOS Pacific Blue (7E.17G9), anti-CD304 PE (Neuropilin-1, NRP1, 3E12), anti-PD-1 PE (29F.1A12), and anti-GARP PE (F011-5). For live/dead discrimination, cells were stained with 7AAD (BD Biosciences, Franklin Lakes, NJ, USA). A blocking step was also performed with anti-CD16/32 mAb (Biolegend) to block FcγRII/III receptors, followed by intracellular staining for cytokines with the Cytofix/Cytoperm kit (BD Biosciences) as per manufacturer instructions. For intracellular cytokine staining, cells were stimulated with 25 ng/mL phorbol 12-myristate 13-acetate (PMA) and 1 μg/mL ionomycin calcium salt (both from Sigma-Aldrich, Merck, St. Louis, MI, USA) for 5 h. The Cytofix/Cytoperm Kit Plus (Golgiplug; BD Biosciences) was also used for cytokine analysis. Stimulated cells were stained with surface markers and then with antibodies against intracellular targets such as anti-IL-17 Pacific Blue (TC11-18H10.1), anti-IFN-γ PE-Cy7 (XMG1.2), anti-Foxp3 PE (MF-14), and anti-Ikzf2 APC (Helios, 22F6) (all from BioLegend). Stained cells were then acquired with an Attune Flow Cytometer (Thermo Fisher Scientific, Waltham, MA, USA) or a FACS ARIAIII (Becton Dickinson, BD, Franklin Lakes, NJ, USA). Analysis of flow cytometry data was performed with FlowJo™ v10.8 software (BD Life Sciences, Franklin Lakes, NJ, USA).

### 2.3. Treg Cell ISOLATION, In-Vitro Stimulation and Suppression Assay

Foxp3+ Tregs cells (CD3^+^ CD4^+^ Foxp3.YFP^+^) were sorted from LNs of *Opn*^fl/fl^*Foxp3*^YFP-Cre^ mice and wild-type controls. For in vitro activation experiments of Foxp3^+^ T cells, sorted cells were cultured with Dynabeads™ mouse T-activator CD3/CD28 (Gibco) at a ratio of 1:1 for 24, 48, and 72 h in the presence of IL-2 (20 IU/mL). For in vitro suppression assays, sorted Foxp3^+^ Tregs from *Opn*^fl/fl^*Foxp3*^YFP-Cre^ and wild-type control mice were cocultured with naïve T cells (CD3^+^ CD4^+^ Foxp3.YFP^-^ CD62L^+^) in ratios 1:1, 1:5, 1:25, and the proliferation of naïve T cells was assessed by flow cytometry (Cell Trace Violet, Thermo Fisher Scientific, C34557) following manufacturer instructions. All samples were analyzed on a FACSAria III cell cytometer (Becton Dickinson, BD). Analysis of flow cytometry data was performed with FlowJo™ v10.8 software.

### 2.4. Cell Transfer Model of Colitis—In Vivo Suppression Assay

For the CD4^+^ T cell transfer model of chronic colitis, naïve T cells (CD4^+^ CD25^−^ CD44^−^ CD62L^+^ CD45RB^high^ T cells) were sorted from B6 mice after enrichment with a CD4^+^ T-Cell Isolation Kit II (Miltenyi Biotec, Bergisch Gladbach, Germany, 130-104-454), according to manufacturer instructions. The following anti-mouse mAbs were used for cell sorting (FACSAria III; Becton Dickinson): anti-CD3 PE-Cy5 (clone 145-2C11), anti-CD4-PE (GK1.5), anti-CD62L Pacific Blue (MEL-14), anti-CD25 APC (PC61.5), anti-CD44 PE-Cy7 (IM7), and anti-CD45RB FITC (C363-16A). Male B6 Rag2^−/−^ recipients were injected i.p. with 5 × 10^5^ sorted T cells. For these experiments, some mice also received wild-type or Opn-deficient Tregs from *Foxp3*^YFP-Cre^ mice or *Opn*^fl/fl^*Foxp3*^YFP-Cre^ mice, respectively, at a ratio of 1 (T reg): 4 (T naïve). Mice were euthanized at 60 days after cell transfer, and mesenteric lymph nodes (MLN) and colon tissues were collected for flow cytometric or histological analysis. Effector T cells from MLNs were isolated with flow cytometry and cultured for 3 days with 2 μg/mL soluble anti-CD3 (OKT3, Functional Grade, eBioscience™, Thermo Fisher Scientific) and 3 μg/mL anti-CD28 (CD28.2, Functional Grade, eBioscience™, Thermo Fisher Scientific). Cytokines were measured in the supernatants of cultured cells using ELISA for IL-10, IL-17a, TNF-α (R&D Systems, Minneapolis, MN, USA), and IFN-γ (BD Biosciences), according to manufacturer instructions. In some experiments, naïve T cells were isolated from CD45.1 congenic mice, allowing for the distinction between donor and recipient cells in subsequent analyses. This approach facilitated tracking and differentiation of the transferred T cells from the host cells in the recipient mice.

### 2.5. Histology and Assessment of Intestinal Inflammation

Mice were euthanized when symptoms of clinical disease (significant weight loss or diarrhea) became apparent in control groups, usually around 8 weeks after the initiation of experiments. Colons were excised for macroscopic damage evaluation and length measurement. Colon tissue was cut vertically or longitudinally, fixed in 10% (vol/vol) buffered formalin, and embedded in paraffin. Four to five microns of paraffin-embedded sections were stained with hematoxylin and eosin, and inflammation was assessed with a modified version of a previously described scoring system [21]. In H&E staining photomicrographs, the original magnification is 20×, with a 100 μm scale bar, unless otherwise stated. The degree of inflammation and the differential cell numbers on microscopic sections were graded blindly. Each sample was graded semi quantitatively from 0 to 3 for the four following criteria: degree of epithelial hyperplasia and goblet cell depletion; leukocyte infiltration in the lamina propria; area of tissue affected; and the presence of markers of severe inflammation such as crypt abscesses, submucosal inflammation, and ulcers. Scores for each criterion were added to give an overall inflammation score for each sample of 0–12. The total colonic score was calculated as the average of the individual scores from the sections of the proximal colon, mid-colon, and distal colon. In the graphs shown, each point corresponds to an individual mouse. Micrographs show sections of mid-colon.

### 2.6. B16-F10 Melanoma Model

For tumor induction, B16-F10 melanoma cells of C57BL/6 background (H-2b) were cultured in complete RPMI for 10 days. Orthotopic tumors (melanomas) were induced in the waxed back skin of B6 Rag2^−/−^ female mice by subcutaneous injection of 105 B16-F10 melanoma cells [22]. This model was also used with adoptive transfers. Briefly, effector T cells (CD3^+^CD4^+^, CD3^+^CD8^+^, Foxp3YFP^-^) from Foxp3^YFP-Cre^ alone or together with Tregs from *Foxp3*^YFP-Cre^ mice or *Opn*^fl/fl^*Foxp3*^YFP-Cre^ mice were transferred to B6 *Rag2*^−/−^ mice at day 0. At day 2, the cells were inoculated with B16-F10 melanoma cells, as described above. Tumor growth in these mice was monitored from days 6 to 18. Tumors were measured with calipers by determining the greatest longitudinal and transverse diameters (length and width), and their volume was calculated as 0.5 × length × width × width. Mice were euthanized when tumors grew larger than 1.100 mm^3^. Lymphocytes from inguinal lymph nodes (ILNs) and tumor-infiltrating lymphocytes (TILs) from melanoma tissue were prepared using a PBS buffer supplemented with 5% FBS and 2 mM EDTA (FACS buffer). Briefly, single cell suspensions from tissues were prepared by passing them through a 40 μm cell strainer (FALCON, Corning, 352340). TILs from melanoma tissue were isolated by dissociating tumor tissue in the presence of collagenase D (1 mg/mL, Roche, Basel, Switzerland) and DNAase I (0.25 mg/mL, Sigma-Aldrich) for 30 min before passing them through a 40 μm cell strainer. Single-cell suspensions were washed twice with FACS buffer, following centrifugation at 350× *g* for 5 min prior to downstream analysis.

### 2.7. Quantitative PCR

Total RNA extraction from pellets of Tregs was performed with the Nucleospin RNA II Kit (Macherey–Nagel, Düren, Germany). For RNA quantification, the Quant-iT RNA Assay Kit (Invitrogen, Waltham, MA, USA) was used. One microgram of RNA was used for each reaction of cDNA synthesis with SuperScript II reverse transcriptase (Invitrogen) and RiboLock RNase inhibitor (Thermo Scientific). Primers were designed (Eurofins MWG, München, Germany) using the Primer3 program.

Foxp3 sense: 5′-CCTCCACTCCACCTAAAG-3′, antisense: 5′-TGAAACCAGACAACTAACAG-3′, *Opn* (*Spp1*) sense: 5′-GGTCAACTAAAGAAGAGGCAA-3′, antisense: 5′-ACAGGAAGAACAGAAGCAAA-G-3′, Ikzf2 sense: 5′-CACCTCAGGACCCATTCTGT-3′, antisense: 5′-TGACAGCGTTCCTTGTGTTC-3′, Tbet sense: 5′-AGCAAGGACGGCGAATGTT-3′, antisense: 5′-GGGTGGACATATAAGCGGTTC-3′, Gata3 sense: 5′GGCCAGGCAAGATGAGAAAG-3′, antisense: 5′-AGGGCGGATAGGTGGTAATG-3′, Rorgt sense: 5′-CCGCTGAGAGGGCTTCAC-3′, antisense 5′-TGCAGGAGTAGGCCACATTACA-3, *ifng* sense: 5′-AAGTGGCATAGATGTGGAAGAA-3′, antisense 5′-GGCTCTGCAGGATTTTCATG-3′, *il17a* sense: 5′-GCCCTCAGACTACCTCAACC-3′, antisense: 5′-CACACCCACCAGCATCTT-3′, *tnfa* sense: 5′-ACACAAGATGCTGGGACAGT-3′, antisense: 5′-CCTACCTTCAGACCTTTCCA-3′, *il6* sense: 5′-GGAAATCGTGGAAATGAGAA-3′, antisense: 5′-TGAAGGACTCTGGCTTTGTC-3′ Real-time PCR was performed with SYBR Green I (Molecular Probes) and Platinum Taq DNA polymerase (Invitrogen) in a StepOnePlus RT-PCR system (Applied Biosystems, Waltham, MA, USA). PCR amplification of the housekeeping gene hypoxanthine phosphoribosyltransferase (Hprt) (sense primer: 5′-GTGAACTGGAAAGCCAAA-3′, antisense primer: 5′-GGACGCAGCAACTGACAT-3′) was performed as a control in each sample reaction.

### 2.8. Statistics

Data were analyzed using the non-parametric unpaired Mann–Whitney U test for statistical analyses of two-group comparisons. Multigroup comparisons were performed using the non-parametric one-way ANOVA Kruskal–Wallis test and Dunn’s multiple comparisons test. Results are presented as mean ± SEM. *p* values < 0.05 were considered statistically significant. Actual *p* values and the number of replicates (n) are reported in each figure legend. Samples were collected and analyzed under the same conditions, and no data were excluded. The data were analyzed using Prism software versions 7.05 and 8.02 (GraphPad). *p* values of 0.05 or less were considered significant.

## 3. Results

### 3.1. Opn-Deficient Foxp3^+^ Tregs Have Impaired In Vivo Function

**Figure 1:**
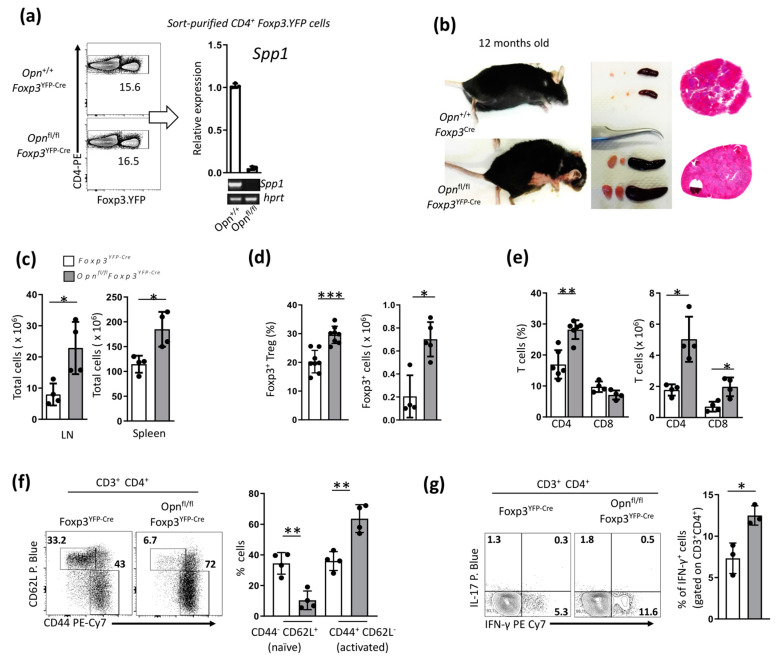
Specific deletion of Opn from Foxp3^+^ Tregs impairs their in vivo function. (**a**) Specific deletion of the *Opn* (*Spp1)* gene in Foxp3-T cells (Opn^fl/fl^ Foxp3^YFP-Cre^). Sorted double-positive CD4^+^ Foxp3. YFP cells were isolated from both *Foxp3*^YFP-Cre^ and *Opn*^fl/fl^ *Foxp3*^YFP-Cre^ mice. Efficient deletion of the *Opn* gene in Opn^fl/fl^Foxp3^YFP-Cre^ mice verified by real-time PCR and expressed as relative expression to the housekeeping gene *Hprt*. (**b**) Signs of lymphoproliferative disorder and skin lesions upon Opn ablation in 12-month-old *Opn*^fl/fl^*Foxp3^YFP-^*^Cre^ mice. A representative hematoxylin and eosin (H&E) stain of spleen and lymph nodes is depicted with evident lymphoproliferation. (**c**) Flow cytometry analysis of total cell counts (× 10^6^) in the lymph nodes and spleen of *Opn*^fl/fl^ *Foxp3*^YFP-Cre^ and *Opn*^+/+^ *Foxp3*^YFP-Cre^ mice. (**d**,**e**) Flow cytometry analysis of total CD4^+^ T cells and Foxp3^+^ Tregs (expressed both as percentage of positive cells and absolute total number) in peripheral lymphoid organs of *Opn*^fl/fl^ *Foxp3*^YFP-Cre^ and *Opn*^+/+^*Foxp3*^YFP-Cre^ mice (n = 8 mice per condition). (**f**) Flow cytometry analysis of CD3^+^CD4^+^ T cells from *Opn*^fl/fl^*Foxp3*^YFP-Cre^ and *Opn*^+/+^ *Foxp3*^YFP-Cre^ mice. Expansion of activated CD44^hi^CD62L^lo^ cells in *Opn*^fl/fl^ *Foxp3*^YFP-Cre^ mice compared with *Opn*^+/+^ *Foxp3*^YFP-Cre^ mice. (**g**) Flow cytometry analysis of the intracellular expression of IL-17 and IFN-γ by CD3^+^CD4^+^ T cells derived from *Opn*^fl/fl^*Foxp3*^YFP-Cre^ and *Opn*^+/+^*Foxp3*^YFP-Cre^ mice (n = 3 mice per condition). Data are representative of three experiments (n = 4 mice, unless otherwise indicated) (**a**–**g**). Non-parametric unpaired Mann–Whitney two-tailed U test, ±SEM; * *p* < 0.05, ** *p* < 0.01, *** *p* < 0.001. LN; lymph nodes, Tregs; T regulatory cells, IL-17; interleukin 17, IFN-γ; Interferon gamma.

To understand the role of Opn expression specifically in Foxp3^+^ Treg cell development and function, we generated mice with Foxp3-specific Opn deletion (*Opn*^fl/fl^ *Foxp3*^YFP-Cre^). This was achieved by crossing mice in which the Opn gene (*Spp1*) was flanked by loxP sites with *Foxp3*^YFP-Cre^ mice (mice expressing yellow fluorescent protein-YFP-Cre recombinase fusion protein under the Foxp3 promoter) [23]. We confirmed the efficient deletion of Opn in *Opn*^fl/fl^ *Foxp3*^YFP-Cre^ mice with sorted CD4^+^ YFP^+^ cells by real-time PCR (Figure 1a). Opn ablation did not affect the frequency of Foxp3^+^ Tregs in the thymus, spleen, and lymph nodes in young mice, and we did not observe any overt signs of pathology in these mice (Supplementary Figure S1a). However, as these mice were aged (10–12 months), we observed signs of lymphoproliferative disorder and skin lesions (Figure 1b,c and Supplementary Figure S1b). We also found elevated numbers of CD4^+^ T cells and, to a lesser extent, CD8^+^ T cells in the peripheral lymphoid organs of *Opn*^fl/fl^ *Foxp3*^YFP-Cre^ mice compared with the controls (Figure 1e). In addition, T cells from *Opn*^fl/fl^*Foxp3*^YFP-Cre^ mice displayed a predominant CD44^hi^CD62L^lo^ activated phenotype and expressed more IFN-γ compared to wild-type control T cells (Figure 1f,g). We also observed increased percentages and absolute numbers of Foxp3^+^ Tregs in *Opn*^fl/fl^ *Foxp3*^YFP-Cre^ mice compared with controls, suggesting that the lymphoproliferative disorder observed in these mice was not due to reduced numbers of Treg cells (Figure 1d).

**Figure 2:**
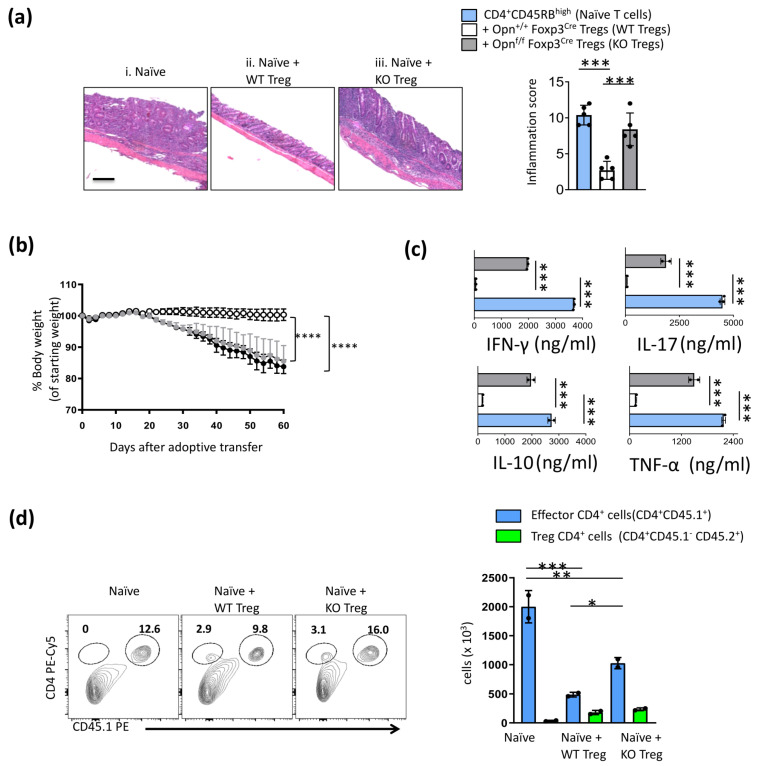
Treg-intrinsic Opn expression is required for optimal Treg suppressive function in an inflammatory colitis model. (**a**) Representative H&E-stained colon sections from (i) *Rag2*^−/−^ mice manifesting inflammatory colitis after adoptive transfer of CD4^+^CD45RB^high^ cells (naïve T cells, blue bars), (Magnification: 20×) (Scale bars: 100 μm) (ii) mice co-transferred with Opn^+/+^ Foxp3^YFP-Cre^ Tregs (WT Tregs, white bars) or (iii) with Opn^fl/fl^ Foxp3^Cre^ Tregs (KO Tregs, grey bars), at a physiologically relevant 1:4 ratio (scale bar: 20 mm). Quantification of histological damage in the colon, expressed as inflammation score in the three mouse groups tested. (**b**) Body weight assessment over 60 days after adoptive transfer of CD4^+^CD45RB^high^ naïve T cells with or without co-transfer of KO Tregs or WT Tregs. (**c**) Cytokine secretion (IFN-γ, IL-17, TNF-α, IL-10) by effector naive T cells isolated from the three mouse subgroups, as assessed by ELISA (all expressed in ng/mL). (**d**) Flow cytometry analysis of naive CD4^+^ T cells (effector CD45.1 T cells) after adoptive transfer with CD45.2 Tregs in *Rag2*^−/−^ mice (numbers of effector T cells and Tregs from mesenteric lymph nodes are indicated by blue and green bars respectively). Data are representative of three experiments (n = 5 mice per condition, unless otherwise indicated) (**a**–**d**). Statistical analysis non-parametric one-way ANOVA Kruskal–Wallis test and Dunn’s multiple comparisons test, ±SEM; * *p* < 0.05, ** *p* < 0.01, *** *p* < 0.001, **** *p* < 0.0001, WT; wild type, KO; knock-out, WT; wild-type controls, IL-17; interleukin 17, IL-10; interleukin 10, IFN-γ; interferon gamma, TNF-a; tumor necrosis factor a.

To examine the effect of Opn deficiency on the suppressive function of Foxp3^+^ Tregs, we performed in vitro suppression assays using Foxp3^+^ Tregs from *Opn*^fl/fl^*Foxp3*^YFP-Cre^ and wild-type control mice. We observed no significant difference between WT and Opn-deficient Tregs in suppressing the proliferation of naïve T cells (Supplementary Figure S1c). Collectively, these data suggest a dispensable role for Opn in regulating the development and in vitro function of Foxp3^+^ Treg cells. However, the Treg activity observed in these assays does not always correlate with in vivo suppression activity; thus, we tested the ability of Opn-deficient Tregs to suppress inflammation in an in vivo model of transfer colitis. In this model, the transfer of CD4^+^ CD25^−^ CD45RB^high^ cells (naïve T cells) into *Rag2*^−/−^ mice leads to inflammatory colitis that can be controlled by the adoptive co-transfer of Tregs. In these experiments, we co-transferred wild-type naïve CD4^+^ T cells and wild-type or Opn-deficient Tregs at a physiologically relevant 1:4 ratio. While mice that received wild-type Tregs were protected from histological damage and weight loss, mice receiving Opn-deficient Tregs failed to limit this inflammatory manifestation (Figure 2a,b). In addition, when we analyzed proinflammatory cytokine secretion (IFN-γ, IL-17, and TNF-α) by effector T cells isolated from these mice, we observed a profound inability of Opn-deficient Tregs to control these responses (Figure 2c). In addition, when we performed similar experiments using congenic markers CD45.1 (for naïve T cells) and CD45.2 (for Tregs), we found that Opn-deficient Tregs failed to control the expansion of naïve T cells as efficiently as WT Tregs (Figure 2d). Finally, we recovered a similar number of Opn deficient Tregs and WT Tregs from the mesenteric lymph nodes in *Rag2*^−/−^ mice, which suggests that the observed differences in these experiments are not due to deficits in trafficking or expansion of these cells following transfer. Collectively, these data suggest that Treg-intrinsic Opn expression is required for optimal Treg suppressive function in vivo.

### 3.2. Opn-Deficient Foxp3^+^ Tregs Exhibit an Unstable Phenotype

**Figure 3:**
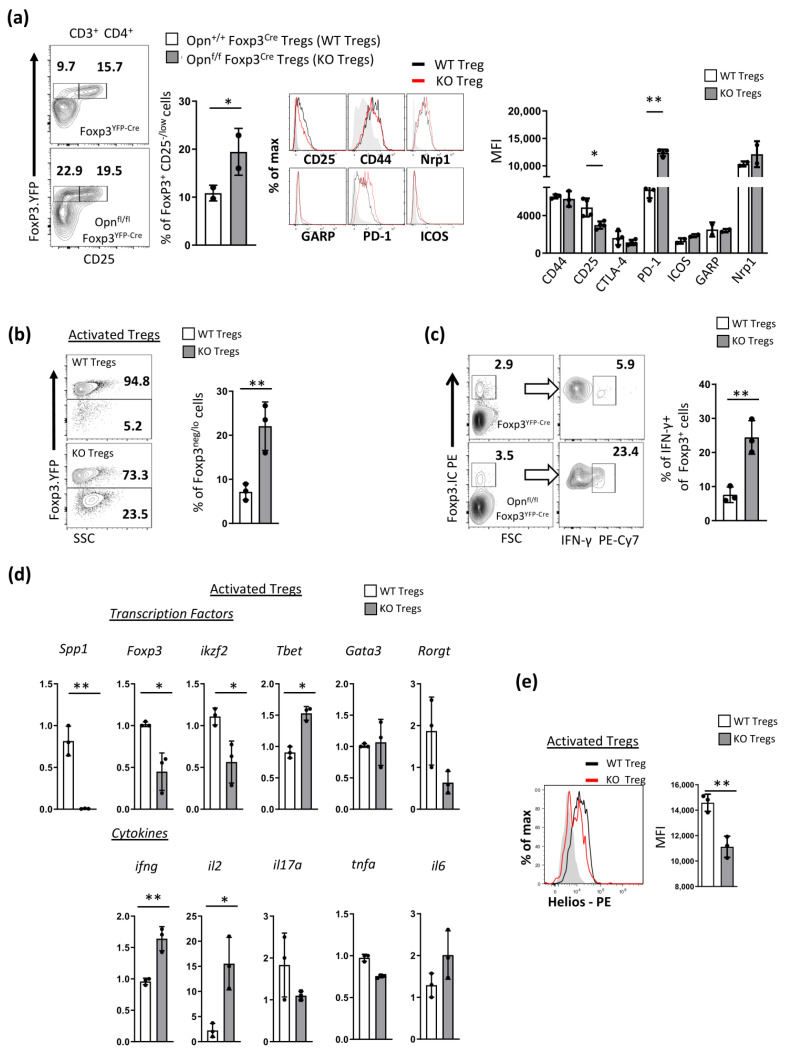
Opn-deficient Foxp3^+^ Tregs exhibit an unstable phenotype. (**a**) Flow cytometry analysis of Foxp3^+^ Tregs from *Opn^f^*^l/fl^*Foxp3*^YFP-Cre^ and control mice (Foxp3^YFP-Cre^). Foxp3 analysis of surface molecules (CD25, CD44, Nrp1, GARP, PD-1, and ICOS). (Middle panel: histogram graphs depicting overlay staining of these markers in Opn-deficient Treg and wild-type Tregs, and right panel: MFI levels Foxp3 from these stainings). (**b**) Flow cytometry analysis of Foxp3 expression in activated Tregs derived from *Opn*^fl/fl^*Foxp3*^YFP-Cre^ and wild-type mice. Foxp3. Activation was induced by stimulation with anti-CD3 and anti-CD28 antibodies in the presence of IL-2 for 72 h. (**c**) Flow cytometry analysis of intracellular IFN-γ expression by activated Tregs from *Opn*^fl/fl^*Foxp3*^YFP-Cre^ and wild-type mice. (**d**) mRNA analysis of gene expression of various transcription factors (Foxp3, ikzf2, Tbet, Gata3, Rorgt) in activated Opn-deficient Tregs and wild-type cells (RT-PCR). Lowe panel: comparative cytokine analysis (IL-2, TNFa, IFN-γ, IL-17a, IL-6) in the culture supernatant of activated Tregs from *Opn*^fl/fl^*Foxp3*^YFP-Cre^ and wild-type mice (by Elisa). (**e**) Flow cytometry analysis of intracellular protein expression of the transcription factor Helios in activated Tregs from *Opn*^fl/fl^*Foxp3*^YFP-Cre^ and wild-type mice. Left panel: overlay histograms of stained Tregs from WT and Opn^fl/fl^Foxp3^YFP-Cre^ mice. Right panel: MFI of Helios expression in Tregs from *Opn*^fl/fl^*Foxp3*^YFP-Cre^ and wild-type mice. Data are representative of three experiments (n = 3 mice per condition) (**a**–**e**). Statistical analysis non-parametric unpaired Mann–Whitney two-tailed U test, ±SEM; * *p* < 0.05, ** *p* < 0.01. Nrp1; neuropil in 1, GARP; glycoprotein A repetitions predominant, PD-1; programmed cell death-1, ICOS; inducible co-stimulator, ikzf2; IKAROS family zinc finger 2, Tbet; T-box expressed in T cells, Gata3; GATA binding protein 3, Rorgt; RAR-related orphan receptor-g, IL-17a; interleukin 17a, IL-10; interleukin 10, IFN-γ; interferon gamma, TNF-a; tumor necrosis factor a, IL-6; interleukin 6, RT-PCR; real time polymerase reaction, MFI; mean fluorescence intensity.

Next, we investigated whether Opn deficiency resulted in phenotypic alterations in Tregs. One of the key hallmark features of stable, bona fide Foxp3^+^ Tregs in non-inflammatory conditions is the high expression of the a-subunit of the IL-2 receptor (CD25). We found that *Opn*^fl/fl^*Foxp3*^YFP-Cre^ mice had elevated numbers of Foxp3^+^ CD25^-/low^ cells compared with wild-type controls, and this was also verified by reduced CD25 MFI values in separate experiments (Figure 3a). We then sort-purified Foxp3^+^ Tregs from *Opn*^fl/fl^*Foxp3*^YFP-Cre^ and control mice and analyzed the expression of other key Treg markers, such as CD44 and Neuropilin-1 (Nrp1), and found no significant differences (Figure 3a). Interestingly, we observed elevated expression of PD-1, which has been described to identify exhausted Tregs infiltrating certain tumors [24] (Figure 3a). In addition, Tregs from *Opn*^fl/fl^*Foxp3*^YFP-Cre^ mice had significantly decreased Foxp3 expression and increased IFNγ expression following in vitro activation (Figure 3b,c). The observed inability of Opn-deficient Tregs to maintain Foxp3 expression was not due to increased apoptosis in these cells (Supplementary Figure S1d).

We next expanded our characterization of Opn-deficient Tregs by looking at the key transcription factor and cytokine expression of these cells following in vitro activation after 72 h. We observed that activated Opn deficient Tregs displayed higher Tbet, IL-2, and IFNɣ gene expression levels compared to wild-type Tregs (Figure 3d). Finally, we found that Opn-deficient Tregs failed to maintain expression of the transcription factor Helios (a key transcriptional marker of Treg stability [25]) following in vitro activation after 72 h at both the mRNA and protein levels (Figure 3d,e). Since it is known that the transcription factor Helios can act as a Foxp3-independent node that regulates Treg stability, we further questioned whether Helios downregulation in Opn-deficient Tregs precedes Foxp3 loss in these cells. We sorted purified Foxp3^+^ Tregs from *Opn*^fl/fl^*Foxp3*^YFP-Cre^ and control mice and activated them with anti-CD3/CD28 beads in vitro at different time points. Interestingly, we found a concomitant downregulation of both at 72 h with no measurable differences at 24 and 48 h (Supplementary Figure S1e).

### 3.3. Mice with Opn-Deficient Foxp3^+^ Tregs Have Enhanced Anti-Tumor Immunity and Reduced Tumor Burden Associated with an Unstable Treg Phenotype

**Figure 4:**
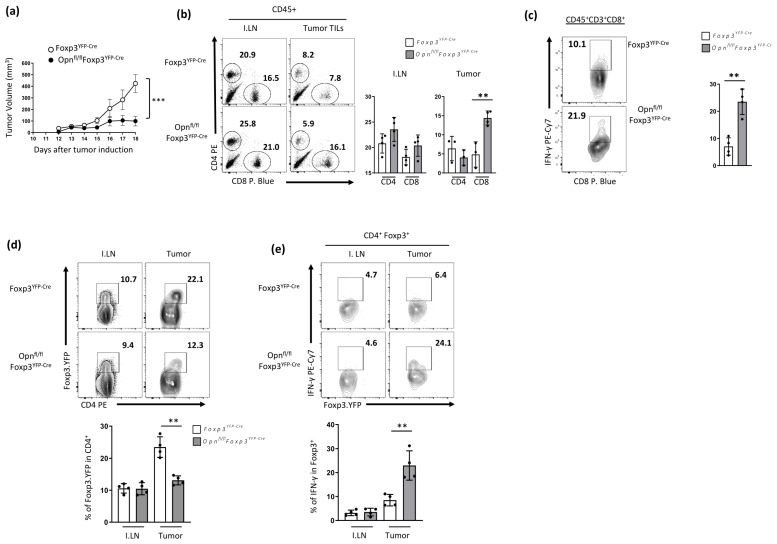
Mice with Opn-deficient Foxp3^+^ Tregs have enhanced anti-tumor immunity and reduced tumor burden associated with an unstable Treg phenotype. (**a**) Assessment of tumor growth (expressed as mm3) in *Opn*^fl/fl^*Foxp3*^YFP-Cre^ and wild-type controls challenged with s. c. inoculation of B16 melanoma cells. (**b**) Flow cytometry analysis of T cell subsets (CD4 and CD8 T cells) in inguinal lymph nodes (I. LN) and tumor-infiltrating lymphocytes (TILs). Foxp3. A representative dot plot of one experiment is shown. Bars represent the percentage of T cell subsets found in I.LN and TILs. (**c**) Flow cytometry analysis of intracellular IFN-γ production by CD8^+^ T cells among TILs from *Opn*^fl/fl^*Foxp3*^YFP-Cre^ and control Foxp3^YFP-Cre^ mice. A representative dot plot of one experiment is shown. Bars represent the percentage of Foxp3.YFP^+^ CD4^+^ T cells in different compartments in the two groups of mice examined. (**d**) Flow cytometry analysis of the percentages of Foxp3 cells expressing CD4 T cells among I.LN and TILs derived from *Opn*^fl/fl^*Foxp3*^YFP-Cre^ and control mice. A representative dot plot from one experiment is shown. Bars represent the percentage of T cell subsets found in I.LN and TILs. (**e**) Flow cytometry analysis of IFN-γ expressing CD4^+^ Foxp3^+^ Tregs from *Opn*^fl/fl^*Foxp3*^YFP-Cre^ and control mice in different compartments. A representative dot plot from one experiment is shown. Bars represent the percentage of IFN-γ-expressing Foxp3^+^ cells found in I.LN and TILs. Data are representative of three experiments (n = 5 mice per condition) (**a**–**e**). Statistical analysis: non-parametric unpaired Mann–Whitney two-tailed U test, ±SEM; ** *p* < 0.01, *** *p* < 0.001. I.LN; inguinal lymph nodes, TILs; tumor-infiltrating lymphocytes.

Thus far, our data suggest that Opn-deficient Tregs display an unstable phenotype that impairs their suppressive function in vivo, especially in inflammatory environments. Interestingly, the observed phenotype resembles the phenotype of *Helios*^/fl^-*Foxp3*^Cre^ mice, presenting signs of lymphoproliferation and elevated IFN-γ expression in conventional T cells [26]. These data allowed us to hypothesize that anti-tumor immunity is affected in these mice. Therefore, we analyzed tumor growth in *Opn*^fl/fl^ *Foxp3*^YFP-Cre^ and wild-type controls challenged with s.c. inoculation of B16 melanoma cells and monitored tumor growth. We found that *Opn*^fl/fl^ *Foxp3*^YFP-Cre^ mice were protected from melanoma tumor development (Figure 4a). Reduced tumor burden was also associated with elevated numbers of tumor-infiltrating CD8^+^ T cells and increased IFN-γ production by these cells in *Opn*^fl/fl^ *Foxp3*^YFP-Cre^ mice compared with TILs from control *Foxp3*^YFP-Cre^ mice (Figure 4b,c). In addition, we analyzed the percentage of Foxp3-expressing CD4^+^ T cells infiltrating B16 melanoma tumors in these mice. We found that the frequency of Foxp3-expressing CD4 T cells in these tumors compared with inguinal lymph nodes (I.LN) was not changed in *Opn*^fl/fl^ *Foxp3*^YFP-Cre^ (≈9–12%) compared with tumors in control mice (≈20–30%) (Figure 4d). Moreover, Tregs from *Opn*^fl/fl^ *Foxp3*^YFP-Cre^ expressed high levels of the pro-inflammatory cytokine IFN-γ compared to wild-type controls, which presented a modest increase (Figure 4e). Collectively, these data suggest that Opn-deficient Tregs present an unstable phenotype, which is associated with enhanced anti-tumor immunity in *Opn*^fl/fl^ *Foxp3*^YFP-Cre^ mice.

### 3.4. Enhanced Anti-Tumor Immunity in Opn^fl/fl^ Foxp3^YFP-Cre^ Is Treg Intrinsic

**Figure 5:**
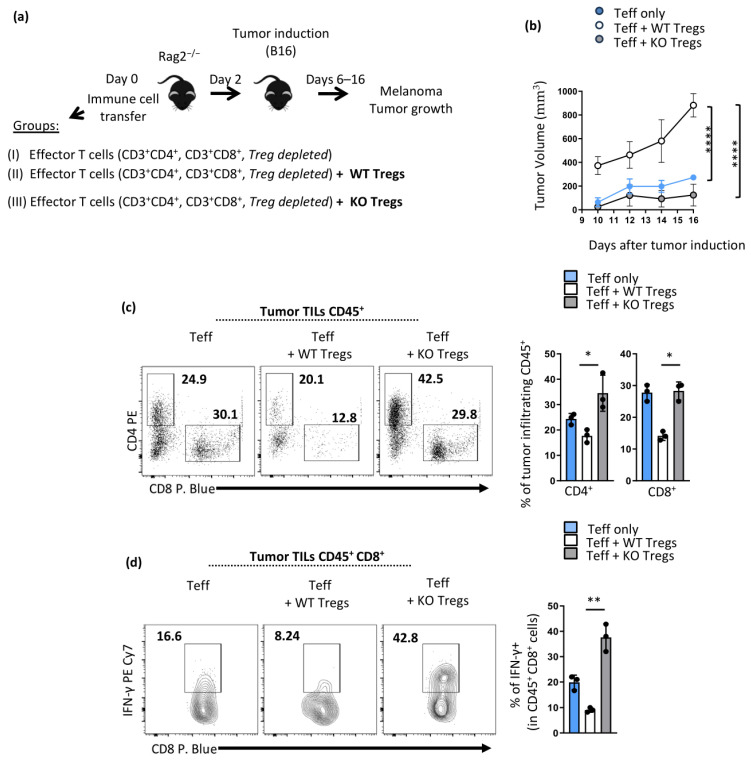
Enhanced anti-tumor immunity in *Opn*^fl/fl^*Foxp3*^YFP-Cre^ mice is Treg intrinsic. (**a**) Schematic illustration of the experimental setup. Purified CD3^+^CD4^+^ and CD3^+^CD8^+^ effector T cells (Foxp3-YFP-; Treg depleted), were transferred into *Rag2*^−/−^ hosts along with Opn-deficient Tregs or wild-type T regs. On day 2, tumor induction (inoculation of B16 melanoma cells) was performed, and tumor growth was monitored in mice from days 6 to 16. (**b**) Tumor development expressed as tumor volume (mm^3^) was monitored for 16 days after tumor induction in the groups of mice presented in 5A (I–III). (**c**) Flow cytometry analysis of T cell subsets (CD4^+^ and CD8^+^) found in TILs in the three mice groups (I–III). A representative dot plot from one of the three experiments is depicted. Bars represent the percentage of tumor-infiltrating CD45^+^ cells, either CD4 or CD8 T cells, in the three mouse groups (I–III). (**d**) Flow cytometry analysis of INF-γ expression by CD8 T cells found in TILs in the three mouse groups studied. A representative dot plot from one experiment is shown. Bars represent the percentage of IFN-γ positive cells (in CD45+ CD8+ cells) in tumor TILs. Data are representative of three experiments (n = 4 mice per condition). Statistical analysis: non-parametric one-way ANOVA Kruskal–Wallis test and Dunn’s multiple comparisons test, ±SEM; * *p* < 0.05, ** *p* < 0.01, **** *p* < 0.0001. TILs; tumor-infiltrating lymphocytes.

To determine whether enhanced anti-tumor immunity in *Opn^f^*^l/fl^ *Foxp3*^YFP-Cre^ mice is Treg intrinsic, we transferred purified CD3^+^CD4^+^ and CD3^+^CD8^+^ effector T cells (T regulatory cells were depleted as we sorted Foxp3^-^YFP-) into *Rag2*^−/−^ hosts along with Opn-deficient Tregs or wild-type controls (Figure 5a). The mice were then inoculated with B16 melanoma cells and monitored for tumor growth. We observed that tumor development in mice that received Opn-deficient Tregs was greatly delayed and halted compared with that in mice that received wild-type Tregs, which presented a more rapid overall tumor burden (Figure 5b). When we analyzed infiltrating lymphocytes in melanoma tumors from these mice, we observed higher numbers of CD4^+^ and CD8^+^ cells in mice that received Opn-deficient Tregs compared with wild-type controls (Figure 5c). In addition, intra-tumoral CD8 effector T cells in hosts transferred with Opn-deficient Tregs displayed increased expression of IFN-γ (Figure 5d).

## 4. Discussion

Studying Foxp3^+^ regulatory T cells (Tregs) is crucial because these cells play a pivotal role in maintaining immune homeostasis, preventing autoimmunity, and modulating the immune response in both inflammatory conditions and cancer [27,28]. Understanding the mechanisms that govern Treg stability and function is essential for developing therapeutic strategies to manage these conditions effectively, particularly in cancer, where the accumulation of these cells within the tumor microenvironment (TME) is often associated with poor prognosis [29]. Our study demonstrates a key role for the protein Opn in controlling Treg cell stability and function. Our results revealed that Opn deficiency specifically in Treg cells impaired their suppressive function over T cell activation, leading to an autoimmune scurfy-like phenotype in aged mice and exacerbated inflammatory colitis, as evident in an in vivo suppression assay of colitis. Moreover, Opn-deficient Tregs presented an unstable phenotype, which was associated with enhanced anti-tumor immunity in mice harboring Opn-deficient Tregs. Although the proinflammatory function of Opn is well documented, here we show that the immunoregulatory role of Opn extends further to include T regulatory cells, which are key regulators of immune responses in inflammatory environments such as the TME.

Our findings indicate that Opn-deficient Tregs exhibit a fragile phenotype, characterized by diminished suppressive capacity and instability. This fragility, a form of Treg plasticity, is consistent with other reports showing that Tregs can become functionally compromised under certain conditions, leading to reduced expression of key markers like Foxp3 [30,31]. Such phenotypic instability has significant implications for immune regulation in cancer and autoimmune diseases, where Treg fragility can alter the balance between immune tolerance and inflammation. Our study demonstrates that Opn-deficient Tregs exhibit several markers indicative of a fragile phenotype. Specifically, these Tregs show reduced expression of CD25, increased expression of PD-1, and higher production of IFN-γ. Upon activation, Opn-deficient Tregs lose their Foxp3 expression, have lower levels of Helios (Ikzf2), and exhibit increased expression of T-bet and IL-2. The molecular relationship between Foxp3 and Helios in Tregs is not yet fully understood, and it remains to be determined whether these two factors are independently impacted in Opn-deficient Tregs. Helios-deficient Tregs are known to develop instability during inflammation, showing reduced Foxp3 expression and increased effector cytokine production, likely due to impaired STAT5 pathway activation. Helios is crucial for regulating IL-2 production by silencing IL-2 gene transcription, thereby maintaining Treg suppressive function. Although Helios loss does not disrupt Treg development, it leads to late-onset autoimmune disease characterized by increased T follicular helper cells, germinal center B cells, and IgG deposition in the kidneys [25,26]. Collectively, these combinations of changes suggest a loss of stability and suppressive function, aligning with the concept of Treg fragility, where Tregs become less effective at maintaining immune tolerance and more prone to adopting an effector-like phenotype.

While our study did not directly investigate the mechanistic basis of these changes, the data strongly suggest that Opn plays a critical role in regulating key pathways involved in the maintenance of Treg stability. Studies from our group and others have shown that Opn is known to interact with the PI3K/Akt, mTOR, and PTEN pathways, which are essential for Treg function [10]. PI3K/Akt signaling, in particular, is vital for sustaining Foxp3 expression and Treg suppressive capacity [32]. A deficiency in Opn could lead to impaired activation of these pathways, contributing to reduced Foxp3 stability and increased expression of pro-inflammatory markers like IFN-γ and T-bet. Additionally, Opn has been shown to influence mTOR signaling, which is critical for Treg metabolic programming and function, and its interaction with TRAF3 further underscores its possible role in regulating Treg effector functions [33,34,35].

Treg infiltration in malignant tumors is known to suppress anti-tumor immune responses, making it crucial to understand the molecular mechanisms that drive tumor immune evasion. While immune checkpoint blockade therapies like anti-CTLA-4 (ipilimumab) and anti-PD-1 (nivolumab) show promise by targeting Foxp3^+^ Tregs in the TME, the exact molecular mechanisms that govern Treg function within tumors remain poorly understood. Opn, typically linked to cancer aggressiveness, plays an unclear role when produced by lymphocytes in the TME. Elevated Opn levels in tumors, including melanoma, are associated with tumor progression and serve as a predictive biomarker [36,37]. In Opn knockout mice, reduced melanoma growth is observed alongside diminished tumor-associated macrophages and Treg accumulation in metastatic sites [34,38]. Our study uniquely demonstrates that Opn deficiency, specifically in Tregs, reduces melanoma progression and is linked to increased CD8^+^ and IFN-γ^+^ tumor-infiltrating lymphocytes. This highlights the intrinsic role of Opn in Tregs and suggests that targeting Opn with cell-specific approaches could enhance the effectiveness of cancer immunotherapies.

It remains uncertain whether the observed effects in Opn-deficient Tregs are driven by the intracellular or extracellular (secreted) forms of Opn, as both forms have been documented to exist in Tregs according to recent studies [39,40]. This distinction is crucial, particularly within the TME, where both forms may contribute to the observed Treg phenotype and the modulation of anti-tumor immunity. The extracellular, secreted form of Opn could play a significant role, especially considering recent findings that show activated or expanded Tregs secreting Opn, which can interact with integrin β1 (Itgb1) to modulate microglia functions in the brain [39]. Albeit different environments, it is plausible to hypothesize that a similar mechanism could occur in the TME, where Itgb1 is known to be expressed by tumor-associated macrophages (TAMs) [41]. The interaction between secreted Opn and Itgb1 on these macrophages could influence their phenotype and function and contribute to the immune evasion observed in tumors. This potential dual role of Opn—both intracellularly within Tregs and extracellularly in modulating the TME—highlights the complexity of its function and underscores the need to consider both forms when interpreting our findings.

While we detailed the effects within the T cell compartment, it is important to acknowledge that Tregs, especially within the TME, interact with other immune cells, such as TAMs, cancer-associated fibroblasts (CAFs), and NK cells, in complex ways. TAMs and CAFs, which are abundant in the TME, express various integrins, including those that act as receptors for Opn, such as integrins αvβ3, α5β1, α9β1, and Itgβ1. These integrins are involved in extracellular matrix (ECM) remodeling, contributing to a microenvironment that supports tumor metastasis [42]. Although our current analysis is limited to Tregs, Opn’s expression within the TME likely influences ECM dynamics and interactions among multiple cell types, such as M1/M2 TAMs. Future studies should explore how Opn deficiency in Tregs might impact or be impacted by these broader immune cell interactions within the TME.

Finally, we also have to acknowledge that the observed phenotypes in the T cell transfer model of melanoma may be partly due to the impaired survival of Opn-deficient Tregs in the TME. While Opn is well known for its pro-survival effects in effector T cells, its role in Treg survival, particularly within the TME, has not been fully explored. This remains an important area for further study to determine whether differential survival contributes to the observed phenotypes in our model.

## 5. Conclusions

Our study is the first to show that Opn deficiency in Foxp3^+^ Tregs impairs their suppressive function in vivo, leading to an unstable phenotype marked by reduced Foxp3 and Helios expression. Notably, mice with Opn-deficient Foxp3^+^ Tregs exhibited enhanced anti-tumor immunity and reduced tumor burden when challenged with B16 melanoma cells. These findings suggest that targeting Opn could improve immunotherapies, with potential benefits in both cancer and inflammatory diseases.

## Data Availability

The data that support the findings of this study are available upon request.

## Supplementary Materials

The following supporting information can be downloaded at: https://www.mdpi.com/article/10.3390/cancers16172952/s1, Figure S1: No difference in the frequency, suppressive activity and viability of Foxp3^+^ Tregs after Opn ablation in *Opn*^fl/fl^*Foxp3*^YFP-Cre^ mice.

## References

[1] Kumari A., Kashyap D., Garg V.K. (2024). Osteopontin in cancer. Adv. Clin. Chem..

[2] Hur E.M., Youssef S., Haws M.E., Zhang S.Y., Sobel R.A., Steinman L. (2007). Osteopontin-induced relapse and progression of autoimmune brain disease through enhanced survival of activated T cells. Nat. Immunol..

[3] Simoes D.C.M., Paschalidis N., Kourepini E., Panoutsakopoulou V. (2022). An integrin axis induces IFN-β production in plasmacytoid dendritic cells. J. Cell Biol..

[4] Uede T. (2011). Osteopontin, intrinsic tissue regulator of intractable inflammatory diseases. Pathol. Int..

[5] Leavenworth J.W., Verbinnen B., Wang Q., Shen E., Cantor H. (2015). Intracellular osteopontin regulates homeostasis and function of natural killer cells. Proc. Natl. Acad. Sci. USA.

[6] Alissafi T., Kourepini E., Simoes D.C.M., Paschalidis N., Aggelakopoulou M., Sparwasser T., Boon L., Hammad H., Lambrecht B.N., Panoutsakopoulou V. (2018). Osteopontin Promotes Protective Antigenic Tolerance against Experimental Allergic Airway Disease. J. Immunol..

[7] Cantor H., Shinohara M.L. (2009). Regulation of T-helper-cell lineage development by osteopontin: The inside story. Nat. Rev. Immunol..

[8] Xanthou G., Alissafi T., Semitekolou M., Simoes D.C., Economidou E., Gaga M., Lambrecht B.N., Lloyd C.M., Panoutsakopoulou V. (2007). Osteopontin has a crucial role in allergic airway disease through regulation of dendritic cell subsets. Nat. Med..

[9] Kourepini E., Aggelakopoulou M., Alissafi T., Paschalidis N., Simoes D.C., Panoutsakopoulou V. (2014). Osteopontin expression by CD103- dendritic cells drives intestinal inflammation. Proc. Natl. Acad. Sci. USA.

[10] Panda V.K., Mishra B., Nath A.N., Butti R., Yadav A.S., Malhotra D., Khanra S., Mahapatra S., Mishra P., Swain B. (2024). Osteopontin: A Key Multifaceted Regulator in Tumor Progression and Immunomodulation. Biomedicines.

[11] Shurin M.R. (2018). Osteopontin controls immunosuppression in the tumor microenvironment. J. Clin. Investig..

[12] Klement J.D., Paschall A.V., Redd P.S., Ibrahim M.L., Lu C., Yang D., Celis E., Abrams S.I., Ozato K., Liu K. (2018). An osteopontin/CD44 immune checkpoint controls CD8+ T cell activation and tumor immune evasion. J. Clin. Investig..

[13] Kim D., Koh J., Ko J.S., Kim H.Y., Lee H., Chung D.H. (2019). Ubiquitin E3 Ligase Pellino-1 Inhibits IL-10-mediated M2c Polarization of Macrophages, Thereby Suppressing Tumor Growth. Immune Netw..

[14] Marson A., Kretschmer K., Frampton G.M., Jacobsen E.S., Polansky J.K., MacIsaac K.D., Levine S.S., Fraenkel E., von Boehmer H., Young R.A. (2007). Foxp3 occupancy and regulation of key target genes during T-cell stimulation. Nature.

[15] Sugimoto N., Oida T., Hirota K., Nakamura K., Nomura T., Uchiyama T., Sakaguchi S. (2006). Foxp3-dependent and -independent molecules specific for CD25^+^CD4^+^ natural regulatory T cells revealed by DNA microarray analysis. Int. Immunol..

[16] Arvey A., van der Veeken J., Samstein R.M., Feng Y., Stamatoyannopoulos J.A., Rudensky A.Y. (2014). Inflammation-induced repression of chromatin bound by the transcription factor Foxp3 in regulatory T cells. Nat. Immunol..

[17] Butti R., Kumar T.V.S., Nimma R., Banerjee P., Kundu I.G., Kundu G.C. (2021). Osteopontin Signaling in Shaping Tumor Microenvironment Conducive to Malignant Progression. Adv. Exp. Med. Biol..

[18] Han H., Ge X., Komakula S.S.B., Desert R., Das S., Song Z., Chen W., Athavale D., Gaskell H., Lantvit D. (2023). Macrophage-derived Osteopontin (SPP1) Protects From Nonalcoholic Steatohepatitis. Gastroenterology.

[19] Das S., Song Z., Han H., Ge X., Desert R., Athavale D., Babu Komakula S.S., Magdaleno F., Chen W., Lantvit D. (2022). Intestinal Osteopontin Protects from Alcohol-induced Liver Injury by Preserving the Gut Microbiome and the Intestinal Barrier Function. Cell Mol. Gastroenterol. Hepatol..

[20] Kourepini E., Paschalidis N., Simoes D.C., Aggelakopoulou M., Grogan J.L., Panoutsakopoulou V. (2016). TIGIT Enhances Antigen-Specific Th2 Recall Responses and Allergic Disease. J. Immunol..

[21] Read S., Malmström V., Powrie F. (2000). Cytotoxic T lymphocyte-associated antigen 4 plays an essential role in the function of CD25^+^CD4^+^ regulatory cells that control intestinal inflammation. J. Exp. Med..

[22] Overwijk W.W., Restifo N.P. (2000). B16 as a mouse model for human melanoma. Curr. Protoc. Immunol..

[23] Rubtsov Y.P., Rasmussen J.P., Chi E.Y., Fontenot J., Castelli L., Ye X., Treuting P., Siewe L., Roers A., Henderson W.R. (2008). Regulatory T cell-derived interleukin-10 limits inflammation at environmental interfaces. Immunity.

[24] Lowther D.E., Goods B.A., Lucca L.E., Lerner B.A., Raddassi K., van Dijk D., Hernandez A.L., Duan X., Gunel M., Coric V. (2016). PD-1 marks dysfunctional regulatory T cells in malignant gliomas. JCI Insight.

[25] Chougnet C., Hildeman D. (2016). Helios-controller of Treg stability and function. Transl. Cancer Res..

[26] Kim H.J., Barnitz R.A., Kreslavsky T., Brown F.D., Moffett H., Lemieux M.E., Kaygusuz Y., Meissner T., Holderried T.A., Chan S. (2015). Stable inhibitory activity of regulatory T cells requires the transcription factor Helios. Science.

[27] Saito T., Nishikawa H., Wada H., Nagano Y., Sugiyama D., Atarashi K., Maeda Y., Hamaguchi M., Ohkura N., Sato E. (2016). Two FOXP3^+^CD4^+^ T cell subpopulations distinctly control the prognosis of colorectal cancers. Nat. Med..

[28] Strauss L., Bergmann C., Szczepanski M.J., Lang S., Kirkwood J.M., Whiteside T.L. (2008). Expression of ICOS on human melanoma-infiltrating CD4^+^CD25highFoxp3^+^ T regulatory cells: Implications and impact on tumor-mediated immune suppression. J. Immunol..

[29] Chaudhary B., Elkord E. (2016). Regulatory T Cells in the Tumor Microenvironment and Cancer Progression: Role and Therapeutic Targeting. Vaccines.

[30] Qiu R., Zhou L., Ma Y., Zhou L., Liang T., Shi L., Long J., Yuan D. (2020). Regulatory T Cell Plasticity and Stability and Autoimmune Diseases. Clin. Rev. Allergy Immunol..

[31] Sakaguchi S., Vignali D.A., Rudensky A.Y., Niec R.E., Waldmann H. (2013). The plasticity and stability of regulatory T cells. Nat. Rev. Immunol..

[32] Huynh A., DuPage M., Priyadharshini B., Sage P.T., Quiros J., Borges C.M., Townamchai N., Gerriets V.A., Rathmell J.C., Sharpe A.H. (2015). Control of PI(3) kinase in Treg cells maintains homeostasis and lineage stability. Nat. Immunol..

[33] Zhao K., Zhang M., Zhang L., Wang P., Song G., Liu B., Wu H., Yin Z., Gao C. (2016). Intracellular osteopontin stabilizes TRAF3 to positively regulate innate antiviral response. Sci. Rep..

[34] Zhang H., Guo M., Chen J.H., Wang Z., Du X.F., Liu P.X., Li W.H. (2014). Osteopontin knockdown inhibits alphav,beta3 integrin-induced cell migration and invasion and promotes apoptosis of breast cancer cells by inducing autophagy and inactivating the PI3K/Akt/mTOR pathway. Cell Physiol. Biochem..

[35] Chang J.H., Hu H., Jin J., Puebla-Osorio N., Xiao Y., Gilbert B.E., Brink R., Ullrich S.E., Sun S.C. (2014). TRAF3 regulates the effector function of regulatory T cells and humoral immune responses. J. Exp. Med..

[36] Moorman H.R., Poschel D., Klement J.D., Lu C., Redd P.S., Liu K. (2020). Osteopontin: A Key Regulator of Tumor Progression and Immunomodulation. Cancers.

[37] Zhao Y., Huang C. (2021). The role of osteopontin in the development and metastasis of melanoma. Melanoma Res..

[38] Sangaletti S., Tripodo C., Sandri S., Torselli I., Vitali C., Ratti C., Botti L., Burocchi A., Porcasi R., Tomirotti A. (2014). Osteopontin shapes immunosuppression in the metastatic niche. Cancer Res..

[39] Shi L., Sun Z., Su W., Xu F., Xie D., Zhang Q., Dai X., Iyer K., Hitchens T.K., Foley L.M. (2021). Treg cell-derived osteopontin promotes microglia-mediated white matter repair after ischemic stroke. Immunity.

[40] Leavenworth J.W., Verbinnen B., Yin J., Huang H., Cantor H. (2015). A p85alpha-osteopontin axis couples the receptor ICOS to sustained Bcl-6 expression by follicular helper and regulatory T cells. Nat. Immunol..

[41] Fei Y., Wu Y., Chen L., Yu H., Pan L. (2024). Comprehensive pan-carcinoma analysis of ITGB1 distortion and its potential clinical significance for cancer immunity. Discov. Oncol..

[42] Kale S., Raja R., Thorat D., Soundararajan G., Patil T.V., Kundu G.C. (2014). Osteopontin signaling upregulates cyclooxygenase-2 expression in tumor-associated macrophages leading to enhanced angiogenesis and melanoma growth via alpha9beta1 integrin. Oncogene.

