# Supplementary material for: Osteopontin Regulates Treg Cell Stability and Function with Implications for Anti-Tumor Immunity and Autoimmunity
Source: Cancers (Basel). 2024 Aug 24;16(17):2952. doi: 10.3390/cancers16172952 (PMC11393878; doi:10.3390/cancers16172952)
Supplement: Supplementary file 1 [file cancers-16-02952-s001.zip › cancers-3154875-supplementary.pdf]

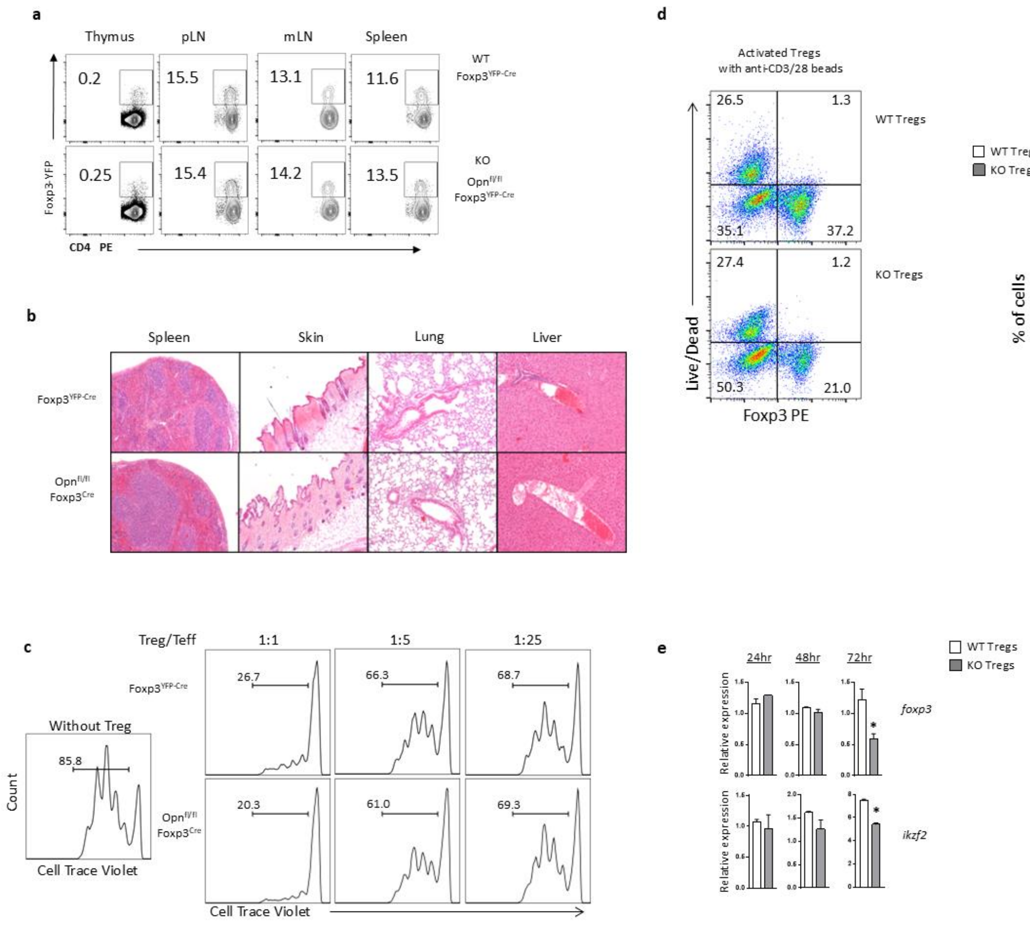

Supplementary Figure S1. No difference in the frequency, suppressive activity and viability of Foxp3+ Tregs after Opn ablation in *Opn*$^{fl/fl}$*Foxp3*$^{YFP-Cre}$ mice. (a) Flow cytometry analysis of Foxp3+ Tregs in the thymus, spleen, and lymph nodes (both mesenteric and peripheral) of young, 6-8 weeks old, mice, after Opn ablation in *Opn*$^{fl/fl}$*Foxp3*$^{YFP-Cre}$ mice (KO). Wild-type (WT) mice, Foxp3$^{YFP-Cre}$, were used as controls. (b) Hematoxylin and eosin (H&E) staining of the spleen, skin, lung, and liver in *Opn*$^{fl/fl}$*Foxp3*$^{YFP-Cre}$ mice aged 10–12 months. (c) WT and Opn-deficient Tregs display similar suppressing ability over the proliferation of naïve T cells. In vitro suppression assay: Effector CD4 T cells (Teff; effectors) were stained with cell trace violet and co-cultured at different ratios (Treg/Teff; 1:5, 1:5, 1:25) with Tregs derived from *Opn*$^{fl/fl}$*Foxp3*$^{YFP-Cre}$ or wild-type mice or without Tregs. (d) Flow cytometric analysis of apoptosis in Tregs derived from *Opn*$^{fl/fl}$*Foxp3*$^{YFP-Cre}$ mice and wild-type controls.. Representative dot plots of Foxp3+ cells stained with live/dead color staining. (e) Expression of Helios and Foxp3 in Opn-deficient Tregs. Sort purified Foxp3+ Tregs from *Opn*$^{fl/fl}$ *Foxp3*$^{YFP-Cre}$ mice and wild-type controls were activated with anti-CD3/CD28 beads in vitro for 24, 48 and 72 hrs. (a-e) Data are representative of three experiments. Unpaired Mann–Whitney two-tailed U test, ±SEM. ** $p < 0.01$, *** $p < 0.001$. pLN; peripheral lymph nodes, mLN; mesenteric lymph nodes, Treg; T regulatory cells, Teff; T effector cells, ikzf2; IKAROS family zinc finger 2, foxp3; forkhead box P3
